# A Method to Combine Neurofilament Light Measurements From Blood Serum and Plasma in Clinical and Population-Based Studies

**DOI:** 10.3389/fneur.2022.894119

**Published:** 2022-06-14

**Authors:** Nicole Rübsamen, Eline A. J. Willemse, David Leppert, Heinz Wiendl, Matthias Nauck, André Karch, Jens Kuhle, Klaus Berger

**Affiliations:** ^1^Institute of Epidemiology and Social Medicine, University of Münster, Münster, Germany; ^2^Neurologic Clinic and Policlinic, Departments of Biomedicine and Clinical Research, MS Center and Research Center for Clinical Neuroimmunology and Neuroscience Basel (RC2NB), University Hospital Basel, University of Basel, Basel, Switzerland; ^3^Department of Neurology With Institute of Translational Neurology, University Hospital Münster, Münster, Germany; ^4^Institute of Clinical Chemistry and Laboratory Medicine, University Medicine Greifswald, Greifswald, Germany; ^5^DZHK (German Centre for Cardiovascular Research), Partner Site Greifswald, University Medicine Greifswald, Greifswald, Germany

**Keywords:** agreement, beta coefficient, biomarker, neurofilament, SiMoA, Z-score standardization

## Abstract

**Introduction:**

Neurofilament light (NfL) can be detected in blood of healthy individuals and at elevated levels in those with different neurological diseases. We investigated if the choice of biological matrix can affect results when using NfL as biomarker in epidemiological studies.

**Method:**

We obtained paired serum and EDTA-plasma samples of 299 individuals aged 37–67 years (BiDirect study) and serum samples of 373 individuals aged 65–83 years (MEMO study). In BiDirect, Passing–Bablok analyses were performed to assess proportional and systematic differences between biological matrices. Associations between serum or EDTA-plasma NfL and renal function (serum creatinine, serum cystatin C, glomerular filtration rate, and kidney disease) were investigated using linear or logistic regression, respectively. All regression coefficients were estimated (1) per one ng/L increase and (2) per one standard deviation increase (standardization using z-scores). In MEMO, regression coefficients were estimated (1) per one ng/L increase of serum or calculated EDTA-plasma NfL and (2) per one standard deviation increase providing a comparison to the results from BiDirect.

**Results:**

We found proportional and systematic differences between paired NfL measurements in BiDirect, i.e., serum NfL [ng/L] = −0.33 [ng/L] + 1.11 × EDTA-plasma NfL [ng/L]. Linear regression coefficients for the associations between NfL and renal function did not vary between the different NfL measurements. In MEMO, one standard deviation increase in serum NfL was associated with greater changes in the outcomes than in BiDirect.

**Conclusion:**

Although there are differences between serum and EDTA-plasma NfL, results can be used interchangeably if standardized values are used.

## Introduction

Neurofilaments are neuronal-specific proteins that consist of a triplet of light (NfL), medium (NfM) and heavy (NfH) chains (1). Analogous to the cardiologist's troponin, NfL is a structural protein that can be detected in the blood at elevated levels in a variety of neurological diseases (2–5). Although a powerful marker across various neurological diseases, the use of NfL in clinical practice and population-based epidemiological studies has been limited to date.

Historically, blood NfL has been measured using enzyme-linked immunosorbent assay (ELISA) and electro-chemiluminescence (ECL) technologies. The analytical sensitivity quantifying NfL in blood has been greatly increased by utilizing the single-molecule assay (Simoa) technology, as compared to the classical ELISA and ECL technologies (6), facilitating studies into NfL as biomarker for a wide range of clinical conditions on a large scale. Studies using serum as well as plasma NfL concentrations are abundant in the literature, but systematic reviews and meta-analyses encountered limitations when combining results from these studies (7–9).

Several research groups have investigated associations between blood NfL and different disease entities by combining patients from two or more cohorts (10–13). Due to differences in sample type (serum or plasma), results were stratified per cohort, which lead to a reduction of statistical power, while ideally one would convert concentrations between serum and plasma and use them combined. Differences between serum and plasma are known for other biomarkers, according to the release of intracellular substances caused by sample clotting (14).

Four studies have investigated if different blood processing protocols could produce consistent results when measuring NfL in the clinical setting with sample sizes ranging from eight to 88 paired plasma (collected in either ethylenediaminetetraacetic acid (EDTA) or lithium heparin tubes) and serum specimens (15–18). They consistently reported higher NfL levels in serum than in plasma. In all studies, however, strengths of correlation were tested via Spearman's correlation coefficient. Correlation describes linear relationship between two sets of data, but not their agreement; it does not quantify the proportional or systematic difference between two methods (19), so it does not allow to derive a formula for conversion between sampling methods.

We aimed to investigate if the choice of biological matrix can affect results when using NfL as biomarker in clinical and population-based epidemiological studies, i.e., if differences in blood processing result in different associations with outcomes of interest. If this was the case, researchers from different studies, who investigate the same outcome, might come to non-consistent conclusions although NfL would have the same biological effect across studies. We measured NfL in two cohort studies (MEMO, BiDirect) and chose commonly used study outcome measures to illustrate differences in regression coefficients, without assuming any causal relationship between NfL and these outcomes.

## Method

### Sample Selection

BiDirect is an observational cohort study that integrates three different cohorts (20). Cohort 3 (reference cohort) includes 912 community-dwelling adults (35–65 years of age) recruited between 2010 and 2013. The participants had been randomly sampled from the population registry of the city of Münster and were followed up every 2 years. Three hundred paired serum and EDTA-plasma samples from the first follow-up examination (2013–2015) were selected in a way to represent a uniform distribution of age and sex in BiDirect. One sample could not be measured for technical reasons.

The Memory and Morbidity in Augsburg Elderly (MEMO) is a 1997/98 re-examination of participants from the 1989/90 World Health Organization Monitoring Trends and Determinants in Cardiovascular Disease (MONICA) Survey Augsburg, Germany (21, 22). Initially in 1989, a random sample of the population of Augsburg, a city in southern Germany, was drawn from the office of registration. The sample's age range was 25–74 years and the response for the MONICA survey was 76.8% (4,940 participants). For the MEMO Study 8 years later, all participants of the MONICA survey aged 65 years and older on July 1, 1997 and living in the city of Augsburg or two large, adjacent suburbs were contacted. The overall response proportion for the MEMO study was 60.6% (385 participants). MEMO samples for 12 participants were not available for NfL measurements.

### Laboratory Measurements

In both cohorts, non-fasting blood samples were collected from each consenting participant. Blood for serum specimens was drawn via venipuncture and collected in clot-activating 9 mL S-monovettes (Sarstedt AG & Co. KG, Nümbrecht, Germany). Samples were processed directly in the study centre within 2 h. They were centrifuged at 2.500 U/min (rpm) for 10 min. After centrifugation, serum and EDTA-plasma aliquots (500 μL) were prepared in 0.5 mL tubes (RNase- and DNase-free, Micronic, Lelystad, The Netherlands), initially stored at −20°C for 5–7 days and then transferred to long-term storage at −80°C. Samples have never been thawed prior to NfL analyses. Processing was done according to the standard operating procedures in BiDirect and MEMO.

In both serum and EDTA-plasma samples, NfL was quantified in 1:4 dilution using the commercial NF-Light Advantage kit (Quanterix, Lexington, MA, USA) applied on the single molecule array HD-X analyser (Quanterix), as previously described (23). In BiDirect, paired serum, and EDTA-plasma samples were consecutively included in the same run to avoid any inter- or intraplate variation influencing the results of the pairwise comparison. Samples were run in duplicate by board-certified technicians blinded to clinical information. The intra-assay and inter-assay coefficients of variation for all samples reported were <15%.

Serum creatinine was measured enzymatically and cystatin C nephelometrically (only in BiDirect) on a dimension vista 1500 analyser, applying commercially available reagents (Siemens Healthcare GmbH, Eschborn, Germany). These assays were performed according to the manufacturer's recommendations. The relevant quality criteria were considered (24).

### Statistical Analysis

We used a Bland–Altman plot (25) and Passing–Bablok regression analysis (26) to compare the biological matrices (299 BiDirect samples) on proportional and systematic differences. The Bland–Altman plot is a simple way to evaluate any bias between the mean differences (27). We used the nonparametric Sfakianakis–Verginis estimator to define an agreement interval (28). Passing–Bablok regression analyses, in contrast to correlation coefficients and Bland–Altman plots, allow to estimate proportional and systematic differences between two measurements and to calculate parameters that allow for correction (19). Passing–Bablok regression analyses assume that measurement errors in both measurements have the same distribution, which not necessarily has to be normal, so we did not log-transform NfL values in these analyses (26). We decided to present formulas to calculate serum NfL from EDTA-plasma NfL because reference values for serum NfL have already been reported (29). As Passing–Bablok regression assumes a linear relationship between two measurements, the cumulative sum linearity test (cusum test) was used to investigate if residuals were randomly distributed above and below the regression line.

We chose commonly used study outcome measures to investigate if differences in blood processing result in different associations. In BiDirect, associations between serum or EDTA-plasma NfL and renal function [serum creatinine, serum cystatin C, glomerular filtration rate estimated via the CKD-EPI equation (30), and self-reported kidney disease (binary outcome)] at baseline (as they were not measured at the first follow-up examination) were investigated using linear or logistic regression models [adjusted for age, sex, and body mass index (31)], respectively. In case of missing outcome or independent variable values, we performed complete-case analyses. NfL was entered as a linear predictor term after confirming linearity of the associations with fractional polynomial analyses (32). Continuous outcome variables were approximately normally distributed so they were not transformed before applying the linear regression analyses. In the first step, regression coefficients and odds ratios (OR) with their corresponding 95% confidence intervals (CI) were estimated using the original NfL values so that effect estimates refer to an increase of one ng/L. In the second step, outcome and independent variables including NfL values were internally standardized using z-scores (33): Per variable, the sample population's mean of that variable was subtracted from each value and results were divided by the sample population's standard deviation (SD). This way, effect estimates refer to an increase of one SD, which makes the effect estimates comparable to each other. To investigate the impact of mixing untransformed NfL values from different biological matrices in a study, we repeated the regression analyses using (1) serum NfL for one half of participants and EDTA-plasma NfL for the other half of participants (randomly drawn) and (2) serum NfL for men and EDTA-plasma NfL for women (in this analysis, we did not adjust for sex). In both analyses, z-score standardization was done separately for serum and EDTA-plasma NfL.

In MEMO, we used the Passing–Bablok formula that was derived in BiDirect to calculate EDTA-plasma NfL values from serum NfL (as there was only serum NfL in MEMO, we had to calculate x using y with the Passing–Bablok formula). Associations between serum NfL and renal function (serum creatinine, glomerular filtration rate estimated via the CKD-EPI equation (30), and glomerular filtration rate ≤ 60 mL/min/1.73 m^2^ as indicator of kidney disease) were investigated using linear or logistic regression models [adjusted for age, sex, and body mass index (31)], respectively. As described above, regression coefficients were estimated (1) per one ng/L increase of serum or calculated EDTA-plasma NfL and (2) per one standard deviation increase, i.e. using standardized outcome and NfL values.

## Results

The BiDirect samples comprised paired sera and EDTA-plasma from 149 women and 150 men aged 37–67 years (Table 1). The MEMO samples comprised sera from 173 women and 200 men aged 65–83 years (Table 1). Serum NfL values were higher in MEMO than in BiDirect because of the different age ranges (Supplementary Figure 1).

**Table 1:** Sample characteristics in the BiDirect and MEMO studies.

**Characteristic**	**BiDirect (*N* = 299)**	**MEMO (*N* = 373)**
Serum NfL [ng/L]	8.6 (6.6, 11.7)* Min: 2.1, Max^‡^: 32.5	15.8 (12.1, 20.4) Min: 5.6, Max: 181.8
EDTA-plasma NfL [ng/L]	8.1 (6.2, 10.8)* Min: 1.9, Max**: 30.6	–
Sex
Men	150 (50%)^†^	200 (54%)
Women	149 (50%)^†^	173 (46%)
Age [years]	53 (46, 60)^†^ Min: 37, Max: 67	73 (69, 76) Min: 65, Max: 83
Body mass index [kg/m^2^]	25.7 (23.4, 28.4)^†^ Min: 17.2, Max: 39.9	27.7 (25.3, 29.8) Min: 18.8, Max: 47.5
*missing*	*1*	*3*
Serum creatinine [mg/dL]	0.86 (0.76, 0.96)^†^ Min: 0.50, Max: 2.66	(0.90, 1.20) Min: 0.20, Max: 3.10
*missing*	*28*	*8*
Serum cystatin C [mg/L]	0.78 (0.70, 0.86)^†^ Min: 0.57, Max: 2.82	–
*missing*	*30*	–
Glomerular filtration rate [mL/min/1.73 m^2^]	91.5 (79.3, 100.4)^†^ Min: 18.2, Max: 123.6	63.1 (51.9, 73.8) Min: 19.3, Max: 161.1
*missing*	*28*	*8*
Glomerular filtration rate ≤ 60 mL/min/1.73 m^2^	5 (2%)^†^	165 (45%)
*missing*	*28*	*8*
Self-reported diagnosis of kidney disease	18 (6%)^†^	–

*Data are median (interquartile range) or n (%).NfL, Neurofilament light chain; MEMO, Memory and Morbidity in Augsburg Elderly; Min, Minimum; Max, Maximum*.

^*^BiDirect follow-up examination (2013–2015).

*^†^BiDirect baseline examination (2010–2013)*.

*^‡^one outlier (128.6 ng/L) excluded*.

**one outlier (106.0 ng/L) excluded.

**Figure 1:**
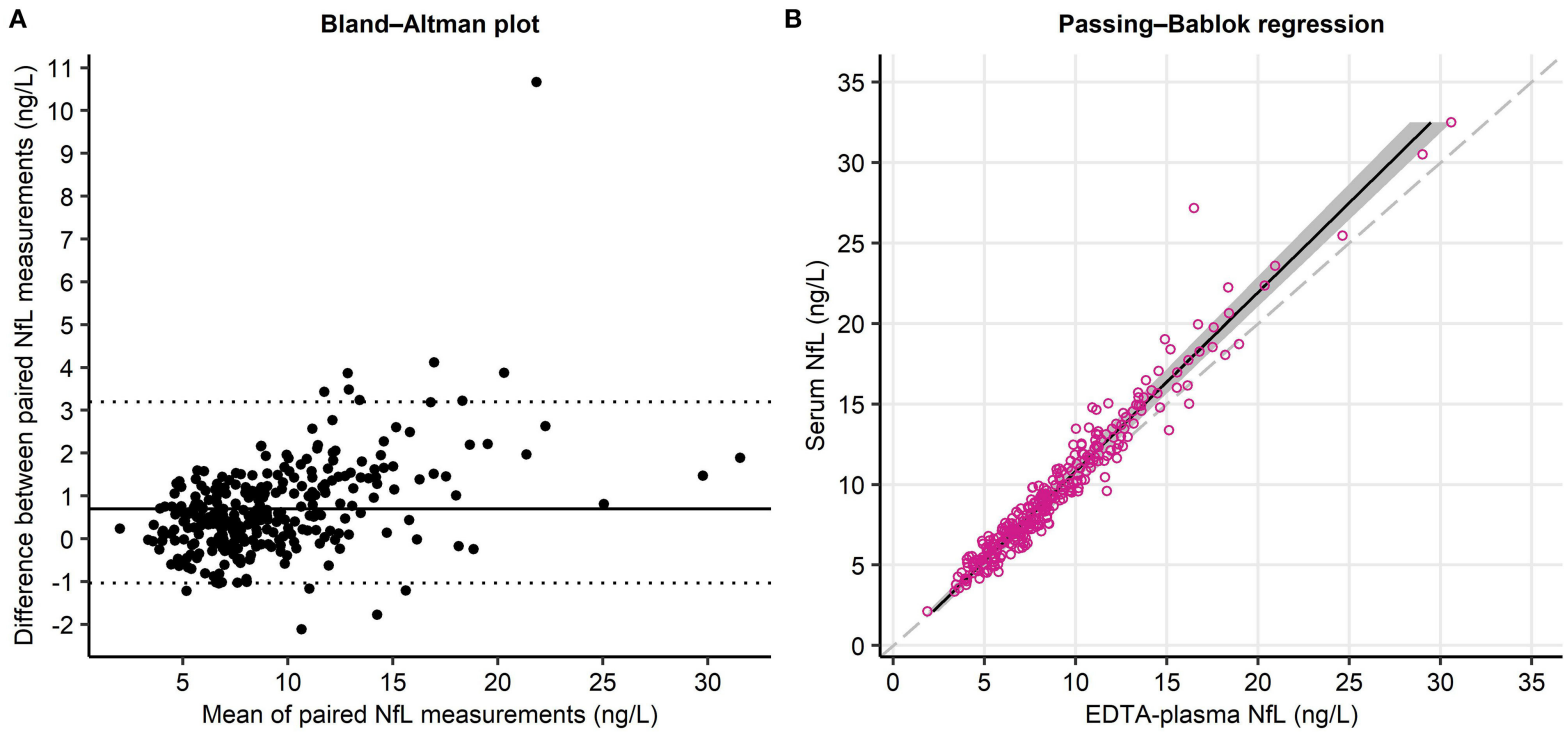
Agreement analyses of NfL measured in different biological matrices. **(A)** Bland–Altman plot: The solid line indicates the mean of the paired differences, the dotted lines represent the Sfakianakis–Verginis limits of agreement. **(B)** Passing–Bablok regression analysis: Red dots indicate individual samples (*n* = 299), the dashed line represents the equation x = y (identity line), and the gray area shows the 95% CIs of the regression line.

In BiDirect, there was a small bias (mean of the paired differences = 0.7; horizontal line in Figure 1A), but overall good agreement (95.3% of the 299 points fell within the Sfakianakis–Verginis limits). The two biological matrices exhibited a proportional difference (slope (95% CI) of regression line 1.12 [1.08; 1.15], as well as a systematic difference (intercept −0.35 [−0.61; −0.07] ng/L). The cusum test indicated a significant deviance from linearity (*p* < 0.001). Exclusion of one outlier (serum NfL level > 120 ng/L) yielded similar regression coefficients (slope: 1.11 [1.08; 1.15], intercept: −0.33 [−0.56; −0.06] ng/L; Figure 1B), but a non-significant cusum test (*p* = 0.082), i.e., no deviance from linearity. Based on these data, the formula for conversion from EDTA-plasma to serum measurements is:


$$\begin{array}{l} \text{serum NfL }[\text{ng/L}]=-\;0.33\;[\text{ng/L}]+1.11\times \text{EDTA}\\ \;-\text{plasma NfL}\;[\text{ng/L}] \end{array}$$

Linear regression coefficients (β) per one ng/L increase for the associations between NfL and continuous variables of renal function (serum creatinine, serum cystatin C, and glomerular filtration rate) did not vary considerably between serum and EDTA-plasma NfL (Table 2), i.e., the absolute differences between the β of serum and EDTA-plasma NfL were <0.1% of the whole ranges (Table 1) of the outcome variables. Odds ratios for kidney disease did also not vary between the different NfL measurements (Table 2).

**Table 2:** Results of regression analyses in BiDirect.

		**β [95% CI] per** **1 ng/L increase**	**|Δ| to serum β**	**β [95% CI] per** **1 SD increase**	**|Δ| to serum β**
Serum creatinine (range: 2.16 mg/dL)	Serum NfL	0.009 [0.005, 0.013]		0.250 [0.139, 0.361]	
	EDTA-plasma NfL	0.010 [0.006, 0.014]	0.001	0.249 [0.139, 0.359]	0.001
	Mixed NfL (random)	0.009 [0.005, 0.013]	0	0.243 [0.134, 0.353]	0.007
	Mixed NfL (by sex)	0.011 [0.007, 0.016]	0.002	0.247 [0.117, 0.378]	0.003
Serum cystatin C (range: 2.25 mg/dL)	Serum NfL	0.006 [0.002, 0.009]		0.214 [0.084, 0.343]	
	EDTA-plasma NfL	0.006 [0.002, 0.010]	0	0.202 [0.074, 0.331]	0.012
	Mixed NfL (random)	0.006 [0.002, 0.009]	0	0.201 [0.073, 0.330]	0.013
	Mixed NfL (by sex)	0.007 [0.003, 0.010]	0.001	0.205 [0.073, 0.336]	0.009
Glome-rular filtration rate (range: 105.4 mL/min/1.73 m^2^)	Serum NfL	−0.753 [−1.129, −0.376]		−0.231 [−0.347, −0.116]	
	EDTA-plasma NfL	−0.839 [−1.260, −0.417]	0.086	−0.228 [−0.342, −0.113]	0.003
	Mixed NfL (random)	−0.801 [−1.189, −0.413]	0.048	−0.228 [−0.342, −0.113]	0.003
	Mixed NfL (by sex)	−0.791 [−1.208, −0.375]	0.038	−0.221 [−0.339, −0.102]	0.01
		**OR [95% CI] per** **1 ng/L increase**	**|Δ| to serum OR**	**OR [95% CI] per** **1 SD increase**	**|Δ| to serum OR**
Kidney disease	Serum NfL	1.05 [0.95, 1.17]		1.26 [0.80, 1.98]	
	EDTA-plasma NfL	1.06 [0.95, 1.19]	0.01	1.28 [0.83, 1.97]	0.02
	Mixed NfL (random)	1.05 [0.94, 1.17]	0	1.25 [0.80, 1.95]	0.01
	Mixed NfL (by sex)	1.06 [0.96, 1.18]	0.01	1.27 [0.82, 1.98]	0.01

*NfL, Neurofilament light chain; SD, Standard deviation; OR, Odds ratio; CI, Confidence interval*.

Standardizing outcomes and independent variables using z-scores revealed that the magnitudes of the associations between NfL and renal function were comparable across all three outcomes: While the β were positive for creatinine and cystatin C, but negative for glomerular filtration rate, the absolute values of the β coefficients ranged between 0.20 and 0.25 per one SD increase of NfL. Again, the differences between the β of serum and EDTA-plasma NfL did not vary considerably. For example, an increase of serum NfL by one SD was associated with a decrease of 0.23 SD in glomerular filtration rate; results for EDTA-plasma NfL were nearly the same. Mixing NfL values from different biological matrices (either original NfL values or internally standardized) resulted in similar regression coefficients as using only NfL values from a single biological matrix (Table 2).

In MEMO, the β and OR per one ng/L increase for the associations between NfL and renal function did also not vary considerably between serum and calculated EDTA-plasma NfL (Table 3). Standardizing outcomes and independent variables using z-scores resulted in identical regression coefficients because calculated EDTA-plasma NfL was just a linear transformation of serum NfL. One SD increase in NfL was associated with greater changes in the outcomes than in BiDirect, e.g., 0.44 SD vs. 0.25 SD increase in serum creatinine in MEMO vs. BiDirect (Table 3).

**Table 3:** Results of regression analyses in MEMO.

		**β [95% CI] per** **1 ng/L increase**	**|Δ| to serum β**	**β [95% CI] per** **1 SD increase**
Serum creatinine (range: 2.90 mg/dL)	Serum NfL	0.010 [0.007, 0.013]		0.442 [0.318, 0.566]
	Calculated EDTA-plasma NfL	0.012 [0.008, 0.015]	0.002	
Glomerular filtration rate (range: 141.8 mL/min/1.73 m^2^)	Serum NfL	−0.464 [−0.637, −0.291]		−0.355 [−0.487, −0.223]
	Calculated EDTA-plasma NfL	−0.516 [−0.709, −0.324]	0.052	
		**OR [95% CI] per 1 ng/L increase**	**|Δ| to serum OR**	**OR [95% CI] per 1 SD increase**
GFR ≤ 60 mL/min/1.73 m^2^	Serum NfL	1.08 [1.04, 1.11]		2.65 [1.71, 4.13]
	Calculated EDTA-plasma NfL	1.08 [1.05, 1.12]	0	

*NfL, Neurofilament light chain; SD, Standard deviation; OR, Odds ratio; CI, Confidence interval*.

## Discussion

This study investigated potential solutions for a better comparability of results based on different biological matrices to harmonize NfL findings across studies. We found proportional and systematic differences between NfL assays, but we show that results were similar when using standardized NfL and outcome values. In consequence, researchers should always report effect estimates that refer to an NfL increase of one SD, in addition to effect estimates that refer to an NfL increase of one measurement unit.

We confirm the numerical differences between serum and EDTA-plasma NfL levels that have been found in other studies (15–18). While these studies focused on the difference between serum and plasma NfL levels based on correlation coefficients, we show (using markers of renal function as examples) that these differences do not persist in regression analyses with NfL as independent variable. In our cohort, serum and EDTA-plasma NfL can even be used interchangeably without affecting the results.

We derived a formula to convert values from EDTA-plasma NfL to serum NfL so that reference values for serum NfL (29) can also be applied if only EDTA-plasma NfL is available. This offers one way to compare results among several studies. In other situations, it might be more convenient to use internally standardized NfL values because this conversion only uses study-specific parameters (mean and SD of NfL). As an example, we report regression coefficients based on data from two different cohort studies. At one glance, these regression coefficients can be compared to each other, revealing greater effect sizes among elderly individuals (MEMO) compared to 37–67-year-old individuals. This direct comparison is not possible without standardizing dependent variables and predictors because standardization removes the influence of different value ranges (34) on the regression coefficients.

The z-score standardization requires the calculation of the mean and standard deviation in a sample. Our results show that these parameters differ between serum and plasma among the same individuals. We, therefore, suggest to separately standardizing serum and plasma values if both are to be used in the same study.

We used markers of renal function as the example outcomes because Akamine et al. (11) have already reported on the relationship between blood NfL concentration and renal function in healthy individuals (based on serum NfL) and patients with diabetes mellitus (based on plasma NfL). They used blood NfL as a dependent variable, included age, sex, BMI, and serum creatinine in separate linear regression analyses. We are aware of this finding and can confirm the positive association between NfL (as dependent variable) and creatinine (as predictor) in our cohorts (data not shown). Our aim, however, was to compare regression coefficients for NfL between different models when only the biological matrix is changed. Therefore, we used NfL as an independent variable as it is usually done in epidemiological studies, e.g., in our previous analysis (35). Our aim was neither to predict renal function based on NfL nor to establish a causal relationship between the variables, but to investigate a cross-sectional association. It has to be kept in mind that renal function was not measured at the first follow-up examination in BiDirect (for which paired sera and EDTA-plasma samples were available), so we used renal function at baseline as outcome solely for illustrative purposes.

There are alternatives to internal standardization when using NfL from different biological matrices as independent variable. Lu et al. (13) combined serum and plasma NfL by using cohort-specific tertile cut-off levels in Cox regression analysis of survival. However, regression coefficients based on categorized independent variables are not comparable across studies. In addition, methods that keep covariates as continuous typically have higher power than methods which use categorization (36).

Although we show that the choice of serum vs. EDTA-plasma does not affect the statistical analyses, researchers have to keep in mind other factors during study planning when deciding whether to measure NfL in serum or in plasma, e.g., logistic constraints. Altmann et al. (18) report that reliability for prolonged storage was slightly better in serum than EDTA-plasma and conclude that native serum samples may be slightly more suitable for shipment or any case of delayed processing.

## Conclusion

Although there are proportional and systematic differences between NfL assays, their results can be used interchangeably if standardized values are used. Our results may help NfL to further evolve as a biomarker in epidemiological studies.

## Data Availability Statement

The raw data supporting the conclusions of this article will be made available by the authors, without undue reservation.

## Ethics Statement

The studies involving human participants were reviewed and approved by Joint Ethics Committee of the University of Münster and the Westphalian Chamber of Physicians. The patients/participants provided their written informed consent to participate in this study.

## Author Contributions

NR: data curation (equal), formal analysis (lead), methodology (lead), visualization (lead), writing—original draft preparation (lead), and writing—review and editing (lead). EW: methodology (equal) and writing—review and editing (equal). DL: writing—review and editing (equal). HW: funding acquisition (equal) and writing—review and editing (equal). MN: data curation (equal), investigation (equal), and writing—review and editing (equal). AK: methodology (equal) and writing—review and editing (equal). JK and KB: conceptualization (equal), data curation (equal), funding acquisition (equal), investigation (equal), and writing—review and editing (equal). All authors contributed to the article and approved the submitted version.

## Funding

MEMO was supported by the German Research Society (Deutsche Forschungsgemeinschaft DFG, grant: BE1996/1-1). BiDirect was funded by research grants (01ER0816 and 01ER1506) from the German Ministry of Research and Education (BMBF). The measurement of NfL was done through funds from the Institute of Epidemiology and Social Medicine and the Department of Neurology, University of Münster and by support of the Swiss National Science Foundation to JK (320030_189140/1).

## Conflict of Interest

The authors declare that the research was conducted in the absence of any commercial or financial relationships that could be construed as a potential conflict of interest.

## Publisher's Note

All claims expressed in this article are solely those of the authors and do not necessarily represent those of their affiliated organizations, or those of the publisher, the editors and the reviewers. Any product that may be evaluated in this article, or claim that may be made by its manufacturer, is not guaranteed or endorsed by the publisher.

## Supplementary Material

Supplementary Figure 1 Distribution of age and NfL in the samples that were selected from two cohort studies.
