# Supplementary figures and images for: A Method to Combine Neurofilament Light Measurements From Blood Serum and Plasma in Clinical and Population-Based Studies
Source: Front Neurol. 2022 Jun 14;13:894119. doi: 10.3389/fneur.2022.894119 (PMC9237479; doi:10.3389/fneur.2022.894119)

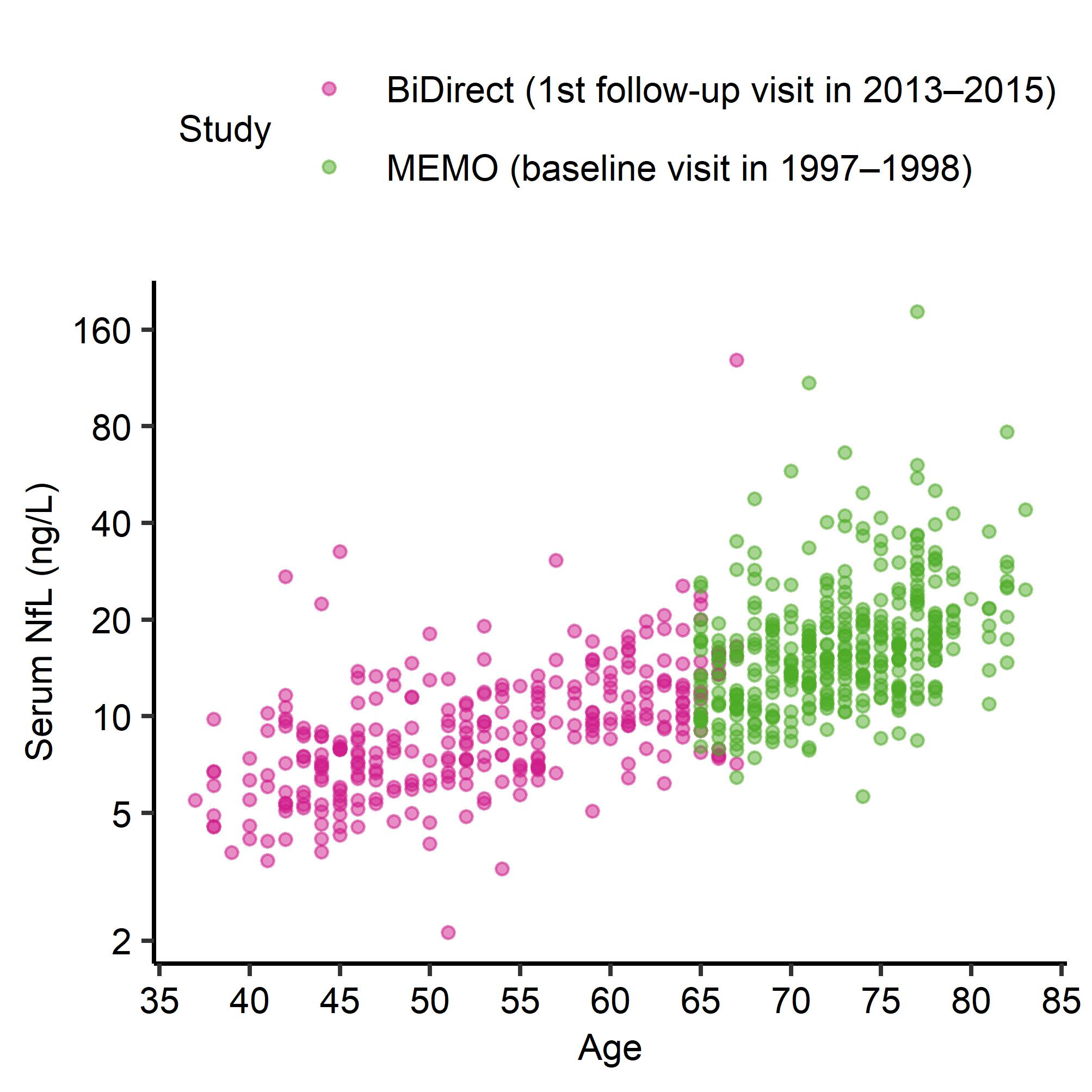

Supplement: Supplementary Figure 1 — Distribution of age and NfL in the samples that were selected from two cohort studies. [file Image_1.JPEG]
